# Artificial Intelligence Tools for Automating Evidence Synthesis: Scoping Review

**DOI:** 10.2196/81597

**Published:** 2026-03-30

**Authors:** Sashika Harasgama, Helen Pearce, Cameron Appel, Liam Loftus, Helena Painter, Isla Kuhn, Justine Karpusheff, Aji Ceesay, John Ford

**Affiliations:** 1 Wolfson Institute of Population Health, Queen Mary University of London, Whitechapel Campus, London, E1 2AD, United Kingdom, +44 (0)20 7882 5555; 2 Medical Library, University of Cambridge, Cambridge, United Kingdom; 3 The Health Foundation, London, United Kingdom

**Keywords:** artificial intelligence, machine learning, large language models, automation, evidence synthesis, systematic reviews as a topic, ChatGPT

## Abstract

**Background:**

Rapidly and accurately synthesizing large volumes of evidence is a time- and resource-intensive process. Once published, reviews often risk becoming outdated, limiting their usefulness for decision makers. Recent advancements in artificial intelligence (AI) have enabled researchers to automate stages of the evidence synthesis process, from literature searching and screening to data extraction and analysis. As previous reviews on this topic have been published, a significant number of tools have been further developed and evaluated. Furthermore, as generative AI increasingly automates evidence synthesis, understanding how it is studied and applied is crucial, given both its benefits and risks.

**Objective:**

This review aimed to map the current landscape of evaluated AI tools used to automate evidence synthesis.

**Methods:**

Following the Joanna Briggs Institute methodology for scoping reviews, we searched Ovid MEDLINE, Ovid Embase, Scopus, and Web of Science in February 2025 and conducted a gray literature search in April 2025. We included articles published in any language from January 2021 onward. Two reviewers independently screened citations using Rayyan, and data were extracted based on study design and key AI-related technical features.

**Results:**

We identified 7841 unique citations through database searches and 19 records through gray literature searching. A total of 222 articles were included in the review. We identified 65 AI tools and 25 open-source models or machine learning (ML) algorithms that automate parts of or the whole evidence synthesis pathway. A total of 54.1% (n=120) of the studies were published in 2024, reflecting a trend toward researching general-purpose large language models (LLMs) for evidence synthesis automation. The most popular tool studied was generative pretrained transformer models, including its conversational interface ChatGPT (n=70, 31.5%). Moreover, 31.1% (n=69) studied tools automated by traditional ML algorithms. No studies compared traditional ML tools to LLM-based tools. In addition, 61.7% (n=137) and 26.1% (n=58) studied AI-assisted automation of title and abstract screening and data extraction, respectively, the 2 most intensive stages and, therefore, amenable to automation. Technical performance outcomes were the most frequently reported, with only 4.1% (n=9) of studies reporting time- or workload-specific outcomes. Few studies pragmatically evaluated AI tools in real-world evidence synthesis settings.

**Conclusions:**

This review comprehensively captures the broad, evolving suite of AI automation tools available to support evidence synthesis, leveraged by increasingly complex AI approaches that range from traditional ML to LLMs. The notable shift toward studying general-purpose generative AI tools reflects how these technologies are actively transforming evidence synthesis practice. The lack of studies in our review comparing different AI approaches for specific automation stages or evaluating their effectiveness pragmatically represents a significant research gap. Optimal tool selection will likely depend on the review topic and methodology and researcher priorities. While they offer potential for reducing workload, ongoing evaluation to mitigate AI bias and to ensure the integrity of reviews is essential for safeguarding evidence-based decision-making.

## Introduction

High-quality evidence synthesis is essential for guiding policy and practice. However, producing such evidence reviews is not only time- and resource-intensive but also challenging to keep up to date due to the volume of studies being published each year. The recent COVID-19 pandemic highlighted the challenges of having accurate, contemporaneous, and rapidly synthesized data, and the opportunities that automation presents in mitigating these [1,2]. Previous studies have estimated that an average systematic review can take approximately 67 weeks to complete [3], often too long for decision makers. The evolving language and text capabilities of artificial intelligence (AI) have increased the scope of automation within the evidence review process, with studies showing that automated tools can help complete systematic reviews in days to weeks [4,5] and significantly reduce workload [6,7].

The advent of evidence synthesis automation was driven by machine learning (ML), the technology that underpins most AI evidence synthesis tools today. ML is a subset of AI that focuses on creating algorithmic systems that can learn from data, recognize patterns, and make decisions, often with model characteristics manually selected by humans [8]. An AI discipline called natural language processing enables computers to understand, interpret, and work with human language (such as written text) and, when used in conjunction with ML, can automate certain language-related tasks [8]. Deep learning is a subset of ML that uses neural networks (computational models that consist of layers of interconnected processing units) to automatically learn more complex patterns from large datasets [8].

Time- and workload-intensive steps within the evidence synthesis pathway, particularly title and abstract screening and data extraction, have been made more efficient through automation tools using machine and deep learning. However, the introduction of generative AI (ie, AI that can autonomously produce text, speech, or other outputs) in the form of commercially available large language models (LLMs), such as ChatGPT (OpenAI) or Claude (Anthropic), has created an opportunity for more creative and complex evidence synthesis automation. Studies are currently trialing LLM-based methods to automate individual stages, such as data extraction, to entire clinical evidence synthesis pipelines [9,10].

LLMs are leveraged by deep learning models called transformers, which use ‘attention’ mechanisms to determine how important each word is in relation to others. They are trained on massive amounts of written data and can capture nuance and context in language more effectively than traditional ML models. Another popular automation approach uses transformers called Bidirectional Encoder Representations from Transformers (BERT), which reads inputs bidirectionally and is pretrained on large sets of text data and then fine-tuned to excel at domain-specific tasks. These newer AI tools may outperform current automation tools in some tasks; however, researchers are still exploring their potential for evidence synthesis, and their performance is yet to be thoroughly validated over time [11,12]. In fact, the use of advanced and generative AI approaches is not without contention and legitimate risks, with the research community fearing loss of academic integrity in the process [11,13,14].

Reviews published to date on this topic have primarily focused on ML tools and approaches used for automating systematic reviews in particular [12,15-17]. For example, Khalil et al [15] performed a scoping review of automation tools for systematic reviews up to mid-2021 and found 10 validated tools that all used ML. Jimenez et al [16] undertook a mapping review of ML tools to assist with systematic reviews and identified 63 tools at the time of publication in December 2022. Khalil et al [18] also conducted a review in 2024 on automation tools for scoping reviews. While these reviews were comprehensive, the rate at which AI technology evolves often means they themselves risk being outdated. A more recent review published by Lieberum et al [12] focused solely on the use of LLMs for performing systematic reviews and found that in half the included studies, LLMs had promising applications, particularly in screening. However, the authors noted that despite the optimism, LLMs are still not quite ready for direct integration into research practice.

The rapid development and likely increasing adoption of generative AI tools to automate evidence synthesis and inform policy and practice warrant a more contemporaneous scoping of the literature. It presents significant risks to the quality and methodological rigor of evidence synthesis, despite its opportunities. Therefore, it is essential to understand the breadth and scope of AI use in this advancing field.

In this scoping review, we aimed to systematically map AI tools available for all types of evidence synthesis, across all its stages, and to describe the current landscape by exploring the underlying automation approaches used by the tools and identifying trends in automated evidence synthesis research.

## Methods

### Ethical Considerations

Ethical approval was not required for this scoping review, as it involved analysis of previously published studies and did not include primary data collection.

### Review Design

We used the Joanna Briggs Institute methodological guidance for scoping reviews [19]. Using the “PCC” mnemonic (population, concept, and context), our review aimed to focus on current AI tools (*P*) used to automate (*C*) the evidence synthesis pathway (*C*). The reporting of this paper was guided by the PRISMA-ScR (Preferred Reporting Items for Systematic Reviews and Meta-Analyses extension for Scoping Reviews) checklist [20], available in Checklist 1. Our review was not registered a priori in PROSPERO.

We aimed to identify studies across steps of the evidence synthesis pathway: (1) searching for the evidence, (2) screening the evidence, (3) extracting the data, (4) assessing the quality of the evidence, (5) analyzing the data, and (6) writing the review. We also aimed to identify which AI methods or techniques were most leveraged for tool development and to determine which parts of the evidence synthesis pathway were the most automated. Our secondary objectives included discussing outcomes typically used to assess performance.

### Search Strategy

The search strategy was adapted from 2 prior reviews’ search strategies [15,16] and iteratively refined through an initial search of Ovid MEDLINE, based on analysis of titles, abstracts, and keywords returned as well as cross-referencing with key articles. The final search was executed across four databases: Ovid MEDLINE, Ovid Embase, Scopus, and Web of Science. We searched for studies published from January 1, 2021, to February 14, 2025, with no language restrictions. Details of our search strategy are available in Part S1 in Multimedia Appendix 1. Our search strategy combined three groups of search terms: (1) evidence review and systematic review terms; (2) AI methods, including ML, deep learning, and other techniques; and (3) terms reflecting the act of automation, such as “support,” “assist,” and “perform.” We also conducted a gray literature search in April 2025 after data extraction of our included studies using Google’s search engine and citation snowballing methods with the tool Litmaps [21] to identify further relevant studies from our included studies.

### Eligibility Criteria

Full details of the eligibility criteria are displayed in Textbox 1. All primary and secondary studies that evaluated the use of AI tools for evidence synthesis in health and care research were included. We did not exclude studies if they did not pertain to health and care research specifically; however, we excluded studies if the AI tool was deemed not transferable to that setting. Studies that did not provide sufficient evaluation of the tool, through technical or other performance-related metrics, were also excluded.

Textbox 1. **Eligibility criteria using the population, intervention, comparison, and outcome (PICO) framework.**

**Inclusion criteria**

- Population and setting
  - Primary or secondary studies that evaluate the use of artificial intelligence (AI) tools for evidence synthesis in health and care research
  - Tool developed in any country
- Intervention
  - AI tools defined as having two characteristics: adaptivity and autonomy (based on the Department for Science, Innovation and Technology guidance)
- Comparison
  - If comparison, to manual research methods
  - If comparison, to other automated tools
- Outcome
  - Any technical or performance-related outcome assessing the AI tool
  - Would be dependent on evidence synthesis task but could include outcomes such as
    - Sensitivity, specificity, precision, and area under the curve
    - Number of relevant studies identified in screening
    - Quality of data extraction
    - Accuracy of meta-analysis
    - Quality and accuracy of writing of evidence synthesis report

**Exclusion criteria**

- Population or setting
  - Studies do not provide any evaluation of AI tools (only descriptions)
- Intervention
  - Automation tools that are not AI or where it is unclear if it is AI

We defined AI according to the regulatory definition provided by the UK Government Department of Science, Innovation and Technology’s policy paper [22]. It defines AI as having two distinct characteristics of adaptivity and autonomy, meaning that AI can continually learn and infer patterns not envisioned by human programmers and can make decisions without the intent or oversight of a human. We excluded studies that discussed automated tools but did not display these characteristics. We also defined evidence synthesis according to the Cochrane definition, which “involves combining information from multiple studies investigating the same topic to comprehensively understand their findings” [23]. No studies were excluded based on comparators or outcomes.

Citations identified through our search were imported and deduplicated using EndNote [24], with Rayyan [25] used to identify further duplicates. Using Rayyan for the entire screening process, records were initially screened via title and abstract by 1 reviewer. Full-text articles were then uploaded and screened for eligibility by another reviewer, with queries or discrepancies resolved by discussion with a third researcher.

### Data Extraction and Synthesis

Data from included studies were extracted into a Microsoft Excel template. Extracted information included baseline study characteristics, such as authors, year of publication, study type, tool name, and outcomes measured. We also extracted specific information regarding the AI method used for tool development, web links to the tools or source code if available, as well as paywall features of the tool if relevant. Consistent with Joanna Briggs Institute scoping review methodology, we did not perform a critical appraisal of the included studies. Findings were thematically synthesized and presented visually, alongside being narratively described with descriptive statistics.

## Results

### Overview

**Figure 1.**
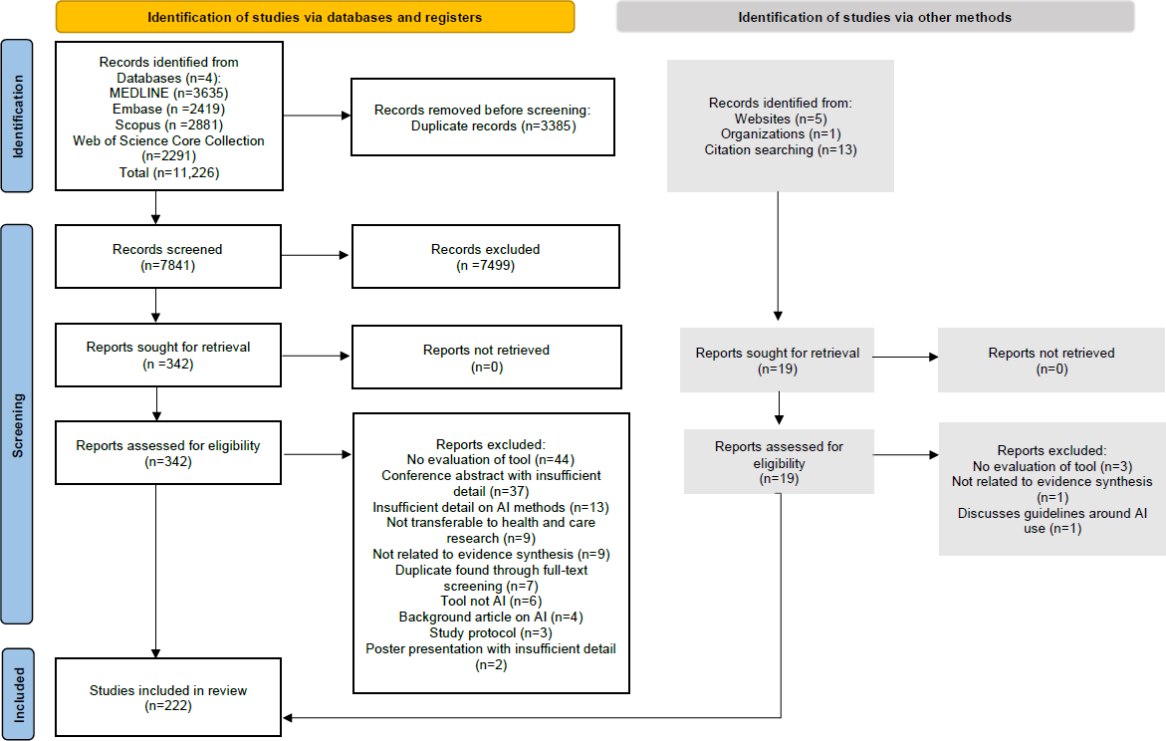
PRISMA (Preferred Reporting Items for Systematic Reviews and Meta-Analyses) flow diagram illustrating the study selection process, including the number of records identified, screened, assessed for eligibility, and included in the final review. AI: artificial intelligence.

A total of 11,226 studies were retrieved from database searches, with 3385 citations removed as duplicates (Figure 1). A total of 7841 citations were screened by their title and abstract, and 7499 were excluded after being deemed irrelevant. The remaining 342 citations underwent full-text screening, and 208 articles were included based on our eligibility criteria. An additional 19 records were identified through snowballing methods and a gray literature search, with 14 records being included. In total, 222 articles were included in the final review.

### Study Characteristics

We included 206 (92.8%) primary studies and 16 (7.2%) secondary studies, with 2 (0.9%) stating they were explicitly AI assisted in their methods [26,27]. Three (1.4%) studies included were written in a language other than English [26,28,29]. A total of 11.7% (26/222) of studies were conference abstracts or posters. We identified 65 (29.3%) distinct AI tools and 25 (11.3%) open-source models or algorithms to automate the evidence synthesis process (Table S3 and S4 in Multimedia Appendix 1). Forty-two (18.9%) of the included studies had no specific tool name or data availability for their model.

Included studies could be divided into three broad categories: (1) methodological studies whereby researchers develop a novel algorithm or model and evaluate its effectiveness for automation; (2) evaluation, diagnostic accuracy, or feasibility studies of existing tools; and (3) comparative studies comparing the performance of AI tools to human researchers or similar tools executing the same task. Ten (4.5%) studies compared the performance of popular general-purpose LLMs, notably ChatGPT, Claude, and Gemini, across various tasks, including screening and writing [30-40]. No studies compared traditional ML tools to LLM-based tools. There was only 1 (0.5%) study that evaluated an AI tool using a randomized trial study design [41].

**Figure 2.**
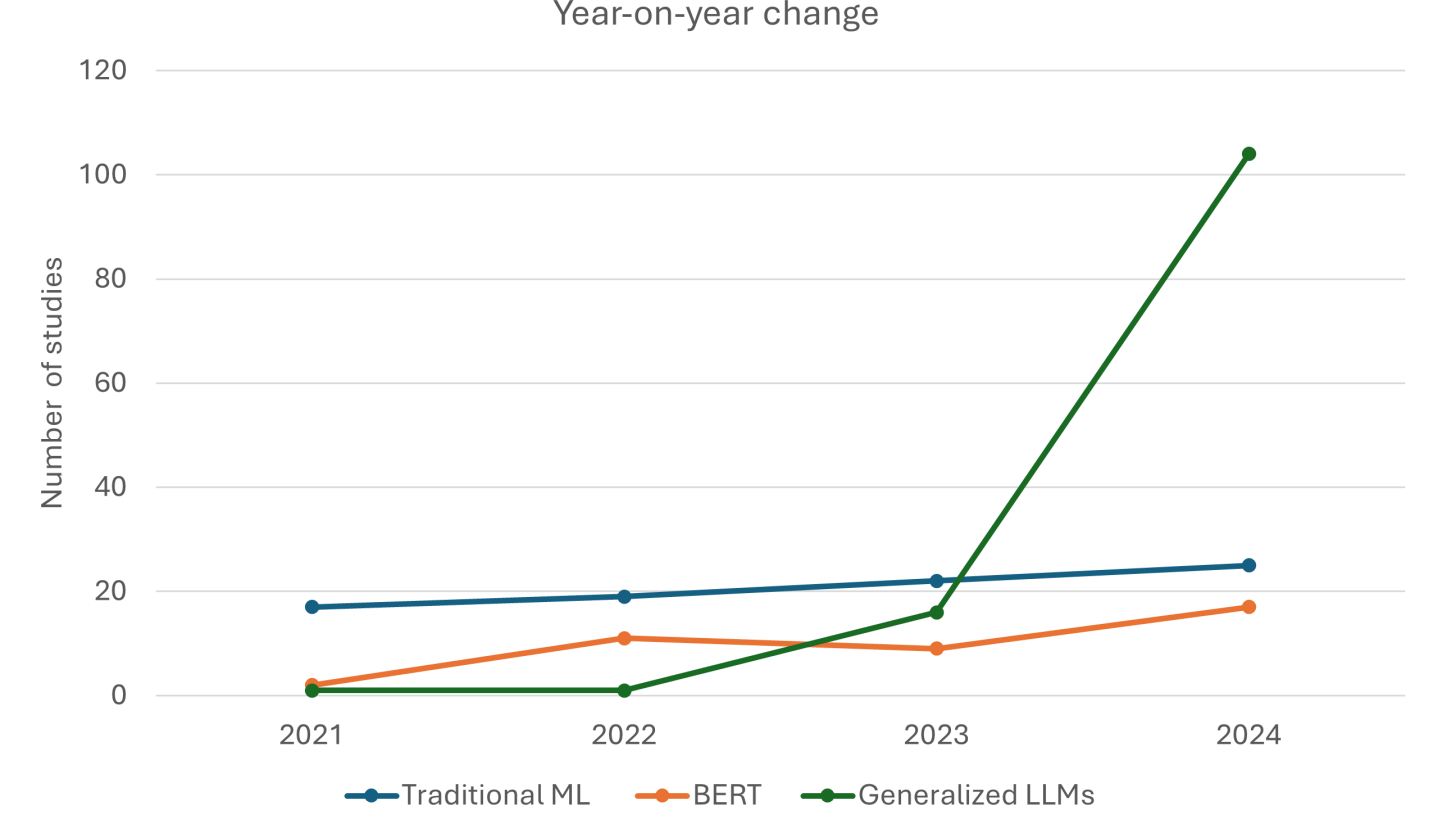
Year-on-year change in the study of three artificial intelligence methods for evidence synthesis automation. BERT: Bidirectional Encoder Representations from Transformers; LLM: large language model; ML: machine learning.

A total of 54.1% (120/222) of included studies were published in 2024, followed by 16.7% (37/222) in 2023 and 14% (31/222) in 2022. Traditional ML, as an automation method, has remained relatively stable across the years, consistently supporting screening and extraction tasks (Figure 2). The use of BERTs has gradually increased since 2021, likely reflecting a growing interest in fine-tuned transformer models for domain-specific tasks. General-purpose LLMs saw a steep increase in 2024, likely coinciding with the widespread availability and maturity of models such as GPT-4 and Claude.

### Evidence Synthesis Pathway

**Figure 3.**
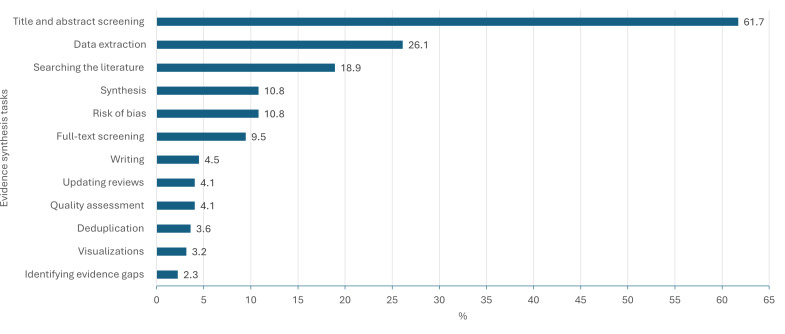
Percentage of studies examining the automation of distinct evidence synthesis stages using artificial intelligence.

Many studies explored multiple areas of the evidence synthesis pathway, with most studies focusing on title and abstract screening (137/222, 61.7%), followed by data extraction (58/222, 26.1%), then literature searching (42/222, 18.9%; Figure 3). The proportion of studies per evidence synthesis stage reflects how amenable it is to automation. We categorized all 65 tools by evidence synthesis stage in Table S5 in Multimedia Appendix 1.

In total, 52.7% (117/222) of included studies researched tools leveraged by transformers. Some examples include general-purpose LLMs such as OpenAI’s generative pretrained transformer (GPT) models or Mistral’s 8×22B, as well as BERT models such as BioBERT or PubMedBERT (pretrained on predominantly biomedical text) that were fine-tuned for specific classification or screening tasks. In addition, 31.1% (69/222) evaluated tools underpinned by traditional ML algorithms alone, such as RCT Tagger, Research Screener, and Abstrackr. Some tools used an ensemble of ML and transformer-based methods, such as ASReview.

### Evolution of AI Methods

**Figure 4.**
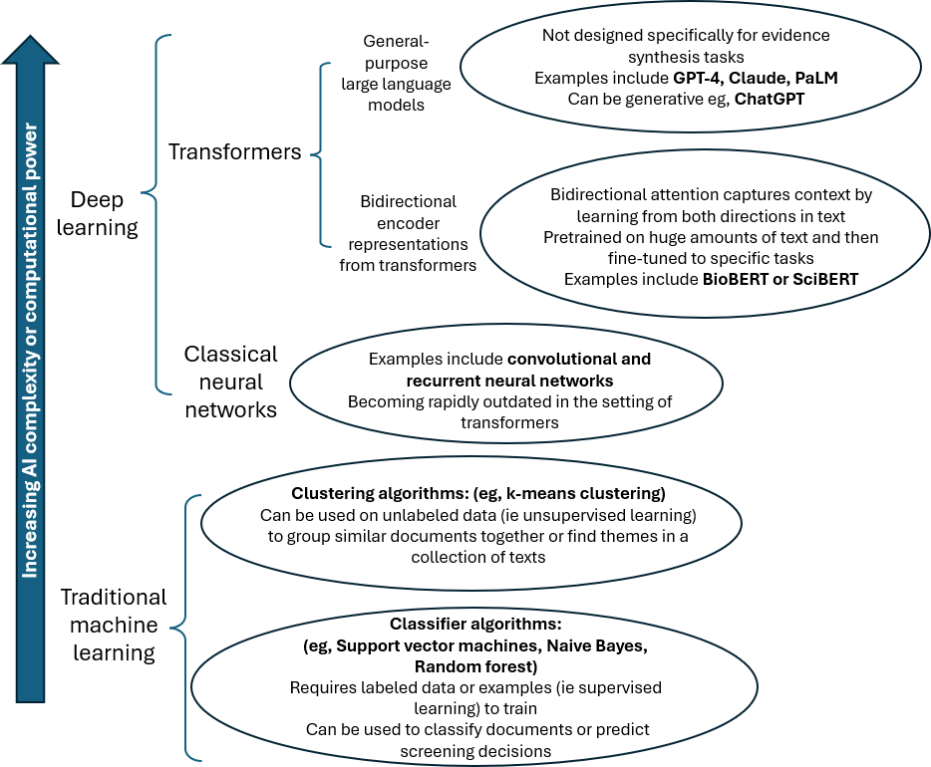
Schematic overview of artificial intelligence methods for evidence synthesis, ranging from traditional machine learning to neural networks and transformer-based large language models. AI: artificial intelligence.

A range of AI approaches were used to automate the evidence synthesis pathway, and these have evolved in computational power or complexity. The schematic in Figure 4 demonstrates two distinct categories of learning: traditional ML using classifier or clustering algorithms and deep learning leveraged by classical neural networks or transformers. Transformers are further divided into their subcategories of BERTs and general-purpose LLMs (ie, not designed specifically for evidence synthesis). Language and text processing abilities of the AI tools increase with complexity and computational power.

**Figure 5.**
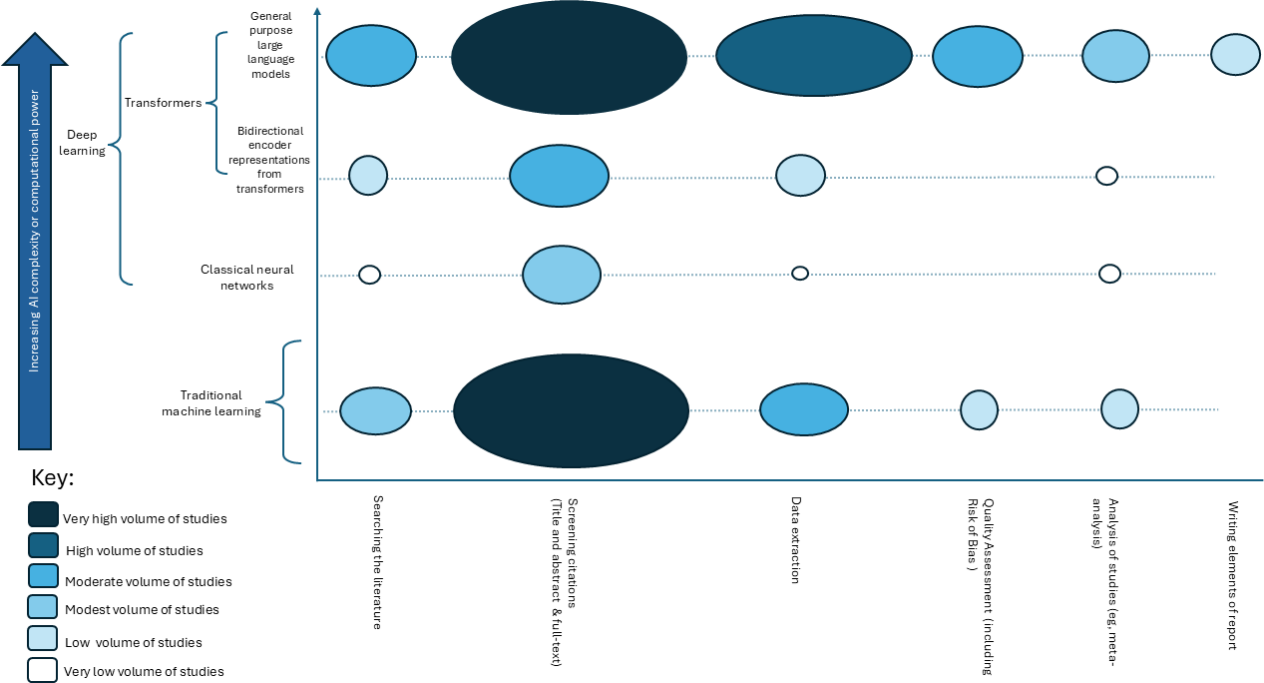
Visualization of included studies depicting the approximate number of studies by evidence synthesis stage and automation method. AI: artificial intelligence.

Both traditional ML and general-purpose LLMs were equally used to automate screening, with a similar distribution noted in data extraction (Figure 5). Traditional ML approaches being frequently leveraged for these stages likely highlight their ongoing suitability for evidence synthesis. Meanwhile, general-purpose LLMs can be applied across the entire evidence synthesis pathway. Generative LLMs are the only AI tool at present capable of producing text and therefore solely represent the automation of writing in evidence synthesis (Figure 5).

### Distribution of Commonly Studied Tools

Of 222 studies, 70 (31.5%) used GPT models or ChatGPT. Other most cited tools included Anthropic’s Claude (16/222, 7.2%), Rayyan (13/222, 5.9%), ASReview (12/222, 5.4%), Abstrackr (9/222, 4.1%), Google’s Gemini (8/222, 3.6%), Covidence (5/222, 2.3%), DistillerSR (6/222, 2.7%), Colandr (3/222, 1.4%), and EPPI Reviewer (2/222, 0.9%). An alphabetized list of tools and their categorization across evidence synthesis tasks is available in Tables S3 and S5 in Multimedia Appendix 1. A list of all open-source models and algorithms is also available in Table S4 in Multimedia Appendix 1.

### Outcomes

The most common outcomes measured were those designed to assess the technical performance of AI tools and models. Sensitivity and recall, which are the same measure, were used in 22.5% (50/222) and 23% (51/222) of studies, respectively. Other frequently reported metrics included specificity, accuracy, precision, area under the curve or area under the receiver operating characteristic curve, and F_1_-score.

Using screening as an example, the area under the curve or the area under the receiver operating characteristic curve measures overall classification performance, indicating how well a model can distinguish between studies for inclusion versus exclusion. The *F*_1_-score measures the balance between precision (how many papers selected by the model were actually relevant) and recall (how many relevant papers were successfully identified).

We also found specific outcomes designed to assess the researchers’ workload or time saved. A popular outcome measure included Work Saved Over Sampling (WSS), which quantifies the percentage of manual screening work that can be avoided by using an AI tool while still maintaining the ability to identify all or most relevant studies. For example, a WSS of 30% means researchers can skip reviewing 30% of the citations while still identifying all relevant studies. Of 222 studies, 9 (4.1%) studies used WSS as a metric to assess the effectiveness of their automated screening tool [42-50].

Agreement between human researchers and AI tools, measured using metrics such as Cohen κ, was also used to assess the validity of AI decisions, although few included studies (12/222, 5.4%) used this approach.

## Discussion

### Principal Findings

This scoping review builds on previous reviews by comprehensively mapping the latest automation tools available across the evidence synthesis pathway and makes an important contribution to understanding how these tools are currently being researched and integrated into evidence synthesis workflows. We identified 65 distinct AI tools, some targeting specific aspects of the evidence synthesis pathway and others covering multiple stages, along with 25 open-source ML algorithms or BERT models designed or fine-tuned for evidence synthesis automation tasks. While published studies have traditionally focused on ML approaches for title and abstract screening and data extraction—the stages most readily automated—recent years have seen a notable shift toward transformer-based approaches for these same stages. Particularly in the past 2 years, there has been a rapid increase in published articles exploring the use of general-purpose LLMs, such as GPT, for evidence synthesis, despite these models not being developed specifically for it. This is notable given the risks generative AI poses due to its training methods and bias. We found that far fewer studies focused on optimizing the search for relevant studies or the analysis of the data or literature.

### What the Results Mean

While transformer-based approaches are expanding the types of complex tasks that can be automated, task-specific ML methods continue to be developed and evaluated for well-defined problems. For example, priority screening using ML-based approaches has been validated thoroughly in the literature [43,51]. On the basis of the results from our review, a wide range of AI tools are being continually deployed and studied at different points across the evidence synthesis pathway, likely reflecting that no one tool or approach is superior to the other and that there is still room for improvement within evidence synthesis automation. While several studies attempted to compare tools with similar underlying AI methods, the lack of studies comparing distinct AI approaches, particularly traditional ML- to LLM-based tools, means we have no clear understanding of their comparative effectiveness.

It is likely that the choice of AI tools will be shaped by the expertise of the evidence synthesized, as well as the purpose and aim of the review itself. Extensively validated traditional ML tools or BERT models fine-tuned to specific research domains may be preferred by researchers or organizations who have a well-defined evidence synthesis task for automation, due to their reliability. For example, researchers conducting systematic reviews of drug or intervention effectiveness may find several well-evaluated tools at their disposal, as most available ML and BERT-based tools excel at interpreting and extracting data from randomized trials with clear eligibility criteria.

General-purpose LLMs, on the other hand, offer flexible language understanding and generative capabilities that can assist researchers in rapidly processing large volumes of text, identifying relevant information, and synthesizing preliminary insights across diverse topics. While these tools may allow for more nuanced interactions with complex evidence, they are likely less acceptable to traditional academic standards.

Most tools identified in this review were designed to optimize systematic reviews, predominantly in the biomedical domain. This is likely because of the very strict methodology that is applied to such reviews, which allows for replicability and, therefore, optimal comparison to the gold standard, manually performed reviews. Limited research explored AI automation in complex, multidisciplinary fields such as public health or social sciences, and few tools addressed nonsystematic review methods, such as narrative, realist, or integrative reviews. This disparity likely reflects a difference in data structure; more standardized data found in quantitative reviews is easier to process and is more machine readable than qualitative data, which tends to be heterogeneous and context dependent.

BERT models continue to be used as a method of automation for screening and data extraction-related tasks, with popular models such as SciBERT and BioBERT being fine-tuned to perform classification tasks for biomedical research questions notably. There is early emerging evidence, however, that general-purpose LLMs are equal if not superior to BERTs at most evidence synthesis tasks [37,52,53]. This potentially indicates to researchers that general-purpose LLMs, even if not designed specifically for evidence synthesis, may be a suitable option for automation if unable to invest the time and resources required to fine-tune BERT models.

Most studies included in our review used technical outcomes to assess tool performance, rather than directly comparing outcomes between AI tools and human researchers performing the same task. While sensitivity and specificity measures are important, there are implications for feasibility if studies do not assess AI tools pragmatically. Adoption of AI tools is likely going to be higher if there is a clear indication that the tool will save a researcher’s time and improve workload for similar outputs.

### Comparison With Previous Literature

Similar to the findings in Khalil and Jimenez’s reviews [15,16], we found that the task most amenable to automation within evidence synthesis was title and abstract screening, with 62% of included studies exploring both ML and deep learning tools to automate this stage. We also found that most ML-based tools identified previously in both reviews remain active and usable today, with several having significant updates incorporating more advanced AI methods such as EPPI Reviewer, Covidence, and Rayyan. Our review was also uniquely able to identify several LLM-based tools that are built specific for evidence synthesis, such as Elicit, Scite, and Consensus. We also found in our review that general-purpose LLMs were predominantly applied to screening and searching of the literature, with GPT being the most frequently cited general-purpose LLM, consistent with Lieberum’s review [12].

Unlike previous reviews, however, which studied ML- and LLM-based tools in isolation, our review demonstrates comparatively the strong shift in the evidence base to studying general-purpose LLMs for evidence synthesis tasks in the last 5 years, particularly for screening, extraction, and, uniquely, writing-related tasks. However, the evidence base for AI-generated or enhanced scientific writing is still evolving and remains a widely debated topic in the research community due to ethical and logistical issues attached to the practice [54-57]. Most journals at present ask for disclosure of AI assistance in the writing process.

Our review also uniquely sought to identify comparative studies between different AI approaches, particularly between traditional ML- and LLM-based tools, as previous reviews focused on distinct approaches in isolation. However, we were unable to identify any such studies within our search time frame, highlighting an important gap in the research. Recent domain-specific evidence has shown that LLM-based tools may outperform ML tools in evidence synthesis tasks such as screening, although these findings are limited due to a lack of generalizability [58].

### Strengths and Limitations

There are several strengths to this scoping review. First, it offers a broad and comprehensive overview of the field, including over 200 studies. We clearly mapped AI tool use across the entire evidence synthesis pathway, providing complete lists of tools available for specific or multiple tasks. It also uses previous reviews’ frameworks to expand on the topic of automation in evidence synthesis, adding consistently to this evidence base. The search strategy was robust, covering multiple academic databases as well as gray literature sources, increasing the likelihood of capturing both peer-reviewed and nontraditional publications.

However, there are several limitations. Given the rapidly evolving nature of AI in evidence synthesis, it is possible that several recent publications were not captured. There is also temporal bias: general-purpose LLMs have only gained widespread adoption in the past 2 years, meaning fewer high-quality peer-reviewed studies are available compared to evaluations of older AI approaches. In addition, this review included studies that explicitly evaluated AI methods or tools to automate evidence synthesis, thereby excluding studies that may have researched AI without clear or proper disclosure of it. This potentially limited our evidence base and underestimated the actual AI usage in evidence synthesis. Additionally, a lack of granularity in reporting the underlying AI methods or technical approaches restricted the meaningful categorization and comparison of tools. Poor reporting of tool development methods also limits generalizability to health and care, particularly when models were trained within specific research domains. Publication bias may also exist, as tools developed in academic settings are more likely to appear in scholarly journals, whereas commercial tools may be underrepresented in the literature. Finally, an inherent limitation of scoping review methodology is the lack of critical appraisal of the evidence base, which limits our understanding of the quality of studies included.

### Recommendations for Policy and Practice

Until we can put in place mitigations that are effective in reducing error and bias in AI tools, there will be a need for a human researcher in the loop [59]. Therefore, the tools highlighted in this paper should be used as adjuncts to humans, rather than replacements, to maintain high-quality and rigorous reviews.

Funders and researchers should continue to support efforts to build an evidence base in this field that can provide a feedback loop for developers to improve upon the tools’ effectiveness, feasibility, and acceptability. In particular, assessing these tools in real-world settings and comparing traditional ML methods to newer LLM-based approaches is pertinent to understanding automation opportunities and challenges. Researchers and funders should foster knowledge sharing on the effective use of emerging AI tools, such as through the International Collaboration of the Automation of Systematic Reviews [60]. This can be through training resources, collaborative learning opportunities, and sharing best practices. This will help build collective understanding and capacity across the research community.

Almost all tools found had paid upgrades or monthly subscriptions. The lack of comprehensive tools that are free to use is concerning from both an accessibility and equity angle and is likely to influence how such tools are integrated into research practice.

Quality standards are needed to ensure ethical tool development, and researchers and stakeholders should adhere to guidelines that stipulate methods to properly and appropriately integrate automation tools into evidence synthesis, such as the recommendations and guidance for Responsible AI in Evidence Synthesis [61].

Decision makers who use AI-assisted evidence syntheses should be aware of the issues around error and bias and should exercise caution when using reviews generated by tools that claim to perform the whole process.

LLMs also have significant electricity demands due to their scale, data requirements, and computational demands [62,63]. Estimates suggest that generative AI processing, from data center operation and cooling, could consume over 8% of the United States’ electricity and 5% of Europe’s by 2030 [64]. Furthermore, manufacturing generative processing units poses significant environmental impacts, particularly for communities near production like in Taichung, Taiwan [64]. While some developers are exploring LLMs using renewable energy, such developments remain in their infancy [65].

In the setting of many organizations and institutions requiring consideration of sustainability in their practice, it is important that we review the environmental implications of LLMs if implemented regularly into evidence synthesis workflows. Therefore, we recommend that sustainability research be undertaken around the use of such AI tools, which is not at all considered in the current evidence base.

### Conclusions

Having accurate, high-quality, and contemporaneous reviews of evidence is essential to making the most informed decisions about health and care. Many of the processes involved in evidence synthesis—particularly identifying relevant studies, screening titles and abstracts, and extracting structured data—involve repetitive, rule-based tasks that are well-suited to automation using AI. With the ongoing development of ML tools and the introduction of LLMs, researchers now have a large suite of AI-based automation tools available to them, as highlighted in our review. While some tools are yet to be comprehensively evaluated, validated, and compared, given the risks generative AI poses to evidence synthesis integrity, the evidence base highlights many potential avenues to improve manual workload. As we continue to integrate such tools into research workflows, it is vital that we continue to monitor outcomes, maintain transparency, and always have a researcher-in-the-loop to ensure that evidence reviews continue to be of a high standard.

## Supplementary material

10.2196/81597 Multimedia Appendix 1 Supplementary materials including detailed database search strategies, PRISMA-S checklist, and categorized tables of AI tools and open-source models identified in the review.

10.2196/81597 Checklist 1 PRISMA-ScR checklist.

## Abbreviations

AI: artificial intelligence
BERT: Bidirectional Encoder Representations from Transformers
GPT: generative pretrained transformer
LLM: large language model
ML: machine learning
PRISMA-ScR: Preferred Reporting Items for Systematic Reviews and Meta-Analyses extension for Scoping Reviews
WSS: Work Saved Over Sampling

## References

[1] Khalil H, Tamara L, Rada G, Akl EA (2022). Challenges of evidence synthesis during the 2020 COVID pandemic: a scoping review. J Clin Epidemiol.

[2] Tercero-Hidalgo JR, Khan KS, Bueno-Cavanillas A (2022). Artificial intelligence in COVID-19 evidence syntheses was underutilized, but impactful: a methodological study. J Clin Epidemiol.

[3] Borah R, Brown AW, Capers PL, Kaiser KA (2017). Analysis of the time and workers needed to conduct systematic reviews of medical interventions using data from the PROSPERO registry. BMJ Open.

[4] Clark J, Glasziou P, Del Mar C, Bannach-Brown A, Stehlik P, Scott AM (2020). A full systematic review was completed in 2 weeks using automation tools: a case study. J Clin Epidemiol.

[5] Clark J, McFarlane C, Cleo G, Ishikawa Ramos C, Marshall S (2021). The impact of systematic review automation tools on methodological quality and time taken to complete systematic review tasks: case study. JMIR Med Educ.

[6] Abogunrin S, Muir JM, Zerbini C, Sarri G (2025). How much can we save by applying artificial intelligence in evidence synthesis? Results from a pragmatic review to quantify workload efficiencies and cost savings. Front Pharmacol.

[7] Rogers K, Miller A, Girgis A, Clark EC, Neil-Sztramko SE, Dobbins M (2025). Leveraging AI to optimize maintenance of health evidence and offer a one-stop shop for quality-appraised evidence syntheses on the effectiveness of public health interventions: quality improvement project. J Med Internet Res.

[8] Ofori-Boateng R, Aceves-Martins M, Wiratunga N, Moreno-Garcia CF (2024). Towards the automation of systematic reviews using natural language processing, machine learning, and deep learning: a comprehensive review. Artif Intell Rev.

[9] Liu J, Lai H, Zhao W (2025). AI-driven evidence synthesis: data extraction of randomized controlled trials with large language models. Int J Surg.

[10] Wang Z, Cao L, Danek B, Jin Q, Lu Z, Sun J (2025). Accelerating clinical evidence synthesis with large language models. NPJ Digit Med.

[11] Clark J, Barton B, Albarqouni L (2025). Generative artificial intelligence use in evidence synthesis: a systematic review. Res synth methods.

[12] Lieberum JL, Toews M, Metzendorf MI (2025). Large language models for conducting systematic reviews: on the rise, but not yet ready for use-a scoping review. J Clin Epidemiol.

[13] Siemens W, von Elm E, Binder H (2025). Opportunities, challenges and risks of using artificial intelligence for evidence synthesis. BMJ Evid Based Med.

[14] Ong AY, Merle DA, Wagner SK, Keane PA (2025). Exploring the dilemma of AI use in medical research and knowledge synthesis: a perspective on deep research tools. J Med Internet Res.

[15] Khalil H, Ameen D, Zarnegar A (2022). Tools to support the automation of systematic reviews: a scoping review. J Clin Epidemiol.

[16] Cierco Jimenez R, Lee T, Rosillo N (2022). Machine learning computational tools to assist the performance of systematic reviews: a mapping review. BMC Med Res Methodol.

[17] Roth S, Wermer-Colan A (2023). Machine learning methods for systematic reviews:: a rapid scoping review. Dela J Public Health.

[18] Khalil H, Pollock D, McInerney P (2024). Automation tools to support undertaking scoping reviews. Res Synth Methods.

[19] Peters MDJ, Marnie C, Tricco AC (2020). Updated methodological guidance for the conduct of scoping reviews. JBI Evid Synth.

[20] Tricco AC, Lillie E, Zarin W (2018). PRISMA extension for scoping reviews (PRISMA-ScR): checklist and explanation. Ann Intern Med.

[21] Litmaps.

[22] A pro-innovation approach to AI regulation. GOV.UK.

[23] (2019). Evidence synthesis - what is it and why do we need it?. Cochrane.

[24] EndNote.

[25] Rayyan OM (2025). AI and the evolution of journalistic practices. J Inf Stud Technol.

[26] Cardoso Sampaio R, Chagas V, Sinimbu Sanchez C (2024). An artificial intelligence (AI)-assisted scoping review of emerging uses of AI in qualitative research and its ethical considerations. Rev Pesq Qual.

[27] Teperikidis E, Boulmpou A, Potoupni V, Kundu S, Singh B, Papadopoulos C (2023). Does the long-term administration of proton pump inhibitors increase the risk of adverse cardiovascular outcomes? A ChatGPT powered umbrella review. Acta Cardiol.

[28] Guo Y, Zhang X, Sun W, Deng H (2024). Application of automated literature screening tools in systematic reviews. Med J Peking Union Med Coll Hosp.

[29] Esposito C, Dell’Omo G, Di Ianni D, Di Procolo P (2024). Human vs. ChatGPT. Is it possible obtain comparable results in the analysis of a scientific systematic review?. Recenti Prog Med.

[30] AlSagri HS, Farhat F, Sohail SS, Saudagar AKJ (2025). ChatGPT or Gemini: who makes the better scientific writing assistant?. J Acad Ethics.

[31] De Cassai A, Dost B, Karapinar YE (2025). Evaluating the utility of large language models in generating search strings for systematic reviews in anesthesiology: a comparative analysis of top-ranked journals. Reg Anesth Pain Med.

[32] Delgado-Chaves FM, Jennings MJ, Atalaia A (2025). Transforming literature screening: the emerging role of large language models in systematic reviews. Proc Natl Acad Sci U S A.

[33] Konet A, Thomas I, Gartlehner G (2024). Performance of two large language models for data extraction in evidence synthesis. Res Synth Methods.

[34] Li M, Sun J, Tan X (2024). Evaluating the effectiveness of large language models in abstract screening: a comparative analysis. Syst Rev.

[35] Omar M, Nassar S, Hijazi K, Glicksberg BS, Nadkarni GN, Klang E (2025). Generating credible referenced medical research: a comparative study of openAI’s GPT-4 and Google’s gemini. Comput Biol Med.

[36] Rathi H, Malik A, Behera DC, Kamboj G (2024). Msr28 use of large language model (LLM) for full-text screening in systematic literature reviews: a comparative analysis. Value Health.

[37] Sanghera R, Thirunavukarasu AJ, Khoury ME (2024). High-performance automated abstract screening with large language model ensembles. arXiv.

[38] Šuster S, Baldwin T, Verspoor K (2024). Zero- and few-shot prompting of generative large language models provides weak assessment of risk of bias in clinical trials. Res Synth Methods.

[39] Thelwall M (2025). Is Google Gemini better than ChatGPT at evaluating research quality?. J. Data Inf. Sci..

[40] Trad F, Charafeddine J, Chkahtoura M, Rahme M, Fuleihan GEH, Chehab A (2024). Streamlining systematic reviews in medical research: a novel application of large language models. J Bone Miner Res.

[41] Arno A, Thomas J, Wallace B, Marshall IJ, McKenzie JE, Elliott JH (2022). Accuracy and efficiency of machine learning-assisted risk-of-bias assessments in “real-world” systematic reviews: a noninferiority randomized controlled trial. Ann Intern Med.

[42] Bravo A, Bennetts L, Atanasov P Accelerating the early identification of relevant studies in title and abstract screening.

[43] Ferdinands G, Schram R, de Bruin J (2023). Performance of active learning models for screening prioritization in systematic reviews: a simulation study into the Average Time to Discover relevant records. Syst Rev.

[44] Yao X, Kumar MV, Su E, Flores Miranda A, Saha A, Sussman J (2024). Evaluating the efficacy of artificial intelligence tools for the automation of systematic reviews in cancer research: a systematic review. Cancer Epidemiol.

[45] Chai KEK, Lines RLJ, Gucciardi DF, Ng L (2021). Research Screener: a machine learning tool to semi-automate abstract screening for systematic reviews. Syst Rev.

[46] van Dinter R, Catal C, Tekinerdogan B (2021). A multi-channel convolutional neural network approach to automate the citation screening process. Appl Soft Comput.

[47] Kusa W, Hanbury A, Knoth P Automation of citation screening for systematic literature reviews using neural networks: a replicability study.

[48] Muthu S (2023). The efficiency of machine learning-assisted platform for article screening in systematic reviews in orthopaedics. Int Orthop.

[49] Akinseloyin O, Jiang XR, Palade V (2024). A question-answering framework for automated abstract screening using large language models. J Am Med Inform Assoc.

[50] Feng Y, Liang S, Zhang Y (2022). Automated medical literature screening using artificial intelligence: a systematic review and meta-analysis. J Am Med Inform Assoc.

[51] Tsou AY, Treadwell JR, Erinoff E, Schoelles K (2020). Machine learning for screening prioritization in systematic reviews: comparative performance of Abstrackr and EPPI-Reviewer. Syst Rev.

[52] Jahan I, Laskar MTR, Peng C, Huang J (2023). Evaluation of ChatGPT on biomedical tasks: a zero-shot comparison with fine-tuned generative transformers. arXiv.

[53] Zhong Q, Ding L, Liu J, Du B, Tao D (2023). Can ChatGPT understand too? A comparative study on ChatGPT and fine-tuned BERT. arXiv.

[54] Draxler F, Werner A, Lehmann F (2024). The AI ghostwriter effect: when users do not perceive ownership of AI-generated text but self-declare as authors. ACM Trans Comput-Hum Interact.

[55] Hosseini M, Gordijn B, Kaebnick GE, Holmes K (2025). Disclosing generative AI use for writing assistance should be voluntary. Res Ethics.

[56] Cohen JF, Moher D (2025). Generative artificial intelligence and academic writing: friend or foe?. J Clin Epidemiol.

[57] Formosa P, Bankins S, Matulionyte R, Ghasemi O (2025). Can ChatGPT be an author? Generative AI creative writing assistance and perceptions of authorship, creatorship, responsibility, and disclosure. AI & Soc.

[58] Dai ZY, Wang FQ, Shen C (2025). Accuracy of large language models for literature screening in thoracic surgery: diagnostic study. J Med Internet Res.

[59] Li Y, Datta S, Rastegar-Mojarad M (2025). Enhancing systematic literature reviews with generative artificial intelligence: development, applications, and performance evaluation. J Am Med Inform Assoc.

[60] International Collaboration for the Automation of Systematic Reviews.

[61] Thomas J, Flemyng E, Noel-Storr A (2024). Responsible AI in Evidence Synthesis (RAISE): guidance and recommendations. Open Science Framework.

[62] Rillig MC, Ågerstrand M, Bi M, Gould KA, Sauerland U (2023). Risks and benefits of large language models for the environment. Environ Sci Technol.

[63] Ji Z, Jiang M (2026). A systematic review of electricity demand for large language models: evaluations, challenges, and solutions. Renew Sustain Energy Rev.

[64] Hosseini M, Gao P, Vivas-Valencia C (2025). A social-environmental impact perspective of generative artificial intelligence. Environ Sci Ecotechnol.

[65] Singh A, Patel NP, Ehtesham A, Kumar S, Khoei TT (2023). A survey of sustainability in large language models: applications, economics, and challenges. arXiv.

