# Supplementary material for: Artificial Intelligence Tools for Automating Evidence Synthesis: Scoping Review
Source: J Med Internet Res. 2026 Mar 30;28:e81597. doi: 10.2196/81597 (PMC13035263; doi:10.2196/81597)
Supplement: Multimedia Appendix 1 [file jmir-v28-e81597-s001.docx]

Supplementary File

# Part S1: Detailed Search Strategy

| Medline via Ovid | 3635 |
| --- | --- |
| Embase via Ovid | 2419 |
| Scopus | 2882 |
| Web of Science Core Collection | 2291 |
| **Total** | **11226** |
| **Total removed at deduplication** | **3339** |
| **Total after deduplication** | **7887** |

Searches run 14^th^ Feb 2025

Limited to 2021 to present

No search filters used

## Medline

Ovid MEDLINE(R) and Epub Ahead of Print, In-Process, In-Data-Review & Other Non-Indexed Citations, Daily and Versions <1946 to February 13, 2025>

1 exp Artificial Intelligence/ 223368

2 exp Data Mining/ 12050

3 exp Pattern Recognition, Automated/ 26813

4 (active learning or (adaptive adj2 algorithm$) or artificial intelligence or AI or artificial narrow intelligence or machine learning or ANI or (automat$ adj2 tool$) or automat$ learning or automat$ classification or automat$ term recognition or backward chaining or clustering engine$ or clustering tool$ or cognitive computing or computational intelligence or computational linguistic$ or computer reasoning or computer vision or connectionist model$ or data crunching or ((text or data) adj2 mining) or datamining or deep learning or document cluster$ or entity annotation$ or entity extraction$ or file cluster$ or forward chaining or generative AI or generative adversarial network$ or GAN or GANs or hierarchical learning or hyperparameter$ or knowledge discovery or knowledge engineering or (learning adj2 algorithm) or (learning adj2 scenario$) or linguistic analy$ or linguistic annotation$ or literature mining or machine intelligence or ((supervised or unsupervised) adj2 learning) or machine perception or machine translation$ or natural language processing or NLP or large language model$ or transformers or Bayesian learning).ti,ab,kw,kf. 352168

5 or/1-4 473701

6 exp "Review Literature as Topic"/ 25428

7 exp Meta-Analysis as Topic/ 31803

8 exp Systematic Reviews as Topic/ 14164

9 (meta-review$ or metareview$ or rapid review$ or scoping review$ or umbrella review$ or evidence review$ or literature review$ or systematic review$ or metaanalys$ or meta-analys$ or ((evidence or research) adj4 synthe$)).ti,ab. 690840

10 or/6-9 705917

11 mt.fs. or method*.ti,kw,kf. or exp Reproducibility of Results/ or exp Research Design/ or (valid* or reliabl* or Opportunit* or challeng* or risk* or support* or assst* or perform* or bias or screening or design* or automi?ation).ti,kw,kf. 7380499

12 5 and 10 and 11 5374

13 limit 12 to yr="2021 -Current" 3635

## Embase

Embase <1974 to 2025 February 13>

1 exp *artificial intelligence/ 63070

2 exp *data mining/ 7036

3 exp *automated pattern recognition/ 10011

4 (active learning or (adaptive adj2 algorithm$) or artificial intelligence or AI or artificial narrow intelligence or machine learning or ANI or (automat$ adj2 tool$) or automat$ learning or automat$ classification or automat$ term recognition or backward chaining or clustering engine$ or clustering tool$ or cognitive computing or computational intelligence or computational linguistic$ or computer reasoning or computer vision or connectionist model$ or data crunching or ((text or data) adj2 mining) or datamining or deep learning or document cluster$ or entity annotation$ or entity extraction$ or file cluster$ or forward chaining or generative AI or generative adversarial network$ or GAN or GANs or hierarchical learning or hyperparameter$ or knowledge discovery or knowledge engineering or (learning adj2 algorithm) or (learning adj2 scenario$) or linguistic analy$ or linguistic annotation$ or literature mining or machine intelligence or ((supervised or unsupervised) adj2 learning) or machine perception or machine translation$ or natural language processing or NLP or large language model$ or transformers or Bayesian learning).ti,ab. 383005

5 or/1-4 409171

6 exp *"systematic review (topic)"/ 2044

7 exp *"meta analysis (topic)"/ 2452

8 (meta-review$ or metareview$ or rapid review$ or scoping review$ or umbrella review$ or evidence review$ or literature review$ or systematic review$ or metaanalys$ or meta-analys$ or ((evidence or research) adj4 synthe$)).ti,ab. 844658

9 or/6-8 844922

10 exp *reproducibility/ 9610

11 exp *methodology/ 605072

12 mt.fs. or method*.ti,kw,kf. or (valid* or reliabl* or Opportunit* or challeng* or risk* or support* or assst* or perform* or bias or screening or design* or automi?ation or "research design").ti,kw,kf. 3438330

13 or/10-12 3916124

14 5 and 9 and 13 3123

15 limit 14 to yr="2021 -Current" 2419

## Scopus

( TITLE ( valid* OR reliabl* OR opportunit* OR challeng* OR risk* OR support* OR assst* OR perform* OR bias OR screening OR design* OR automi?ation OR reproducib* OR method* ) ) OR ( KEY ( valid* OR reliabl* OR opportunit* OR challeng* OR risk* OR support* OR assst* OR perform* OR bias OR screening OR design* OR automi?ation OR reproducib* OR method* ) OR ( ABS ( valid* OR reliabl* OR opportunit* OR challeng* OR risk* OR support* OR assst* OR perform* OR bias OR screening OR design* OR automi?ation OR reproducib* ) ) ) AND ( ( TITLE ( ( "meta-review*" OR "metareview*" OR "rapid review*" OR "scoping review*" OR "umbrella review*" OR "evidence review*" OR "literature review*" OR "systematic review*" OR "metaanalys*" OR "meta-analys*" OR ( ( evidence OR research ) W/4 synthe* ) ) ) ) OR ( KEY ( ( "meta-review*" OR "metareview*" OR "rapid review*" OR "scoping review*" OR "umbrella review*" OR "evidence review*" OR "literature review*" OR "systematic review*" OR "metaanalys*" OR "meta-analys*" OR ( ( evidence OR research ) W/4 synthe* ) ) ) ) ) AND ( TITLE-ABS-KEY ( "active learning" OR ( "adaptive" W/2 "algorithm*" ) OR "artificial intelligence" OR ai OR "artificial narrow intelligence" OR ani OR ( "automat*" W/2 "tool*" ) OR "automat* learning" OR "automat* classification" OR "automat* term recognition" OR "backward chaining" OR "clustering engine*" OR "clustering tool*" OR "cognitive computing" OR "computational intelligence" OR "computational linguistic*" OR "computer reasoning" OR "computer vision" OR "connectionist model*" OR "data crunching" OR ( ( text OR data ) W/2 mining ) OR "datamining" OR "deep learning" OR "document cluster*" OR "entity annotation*" OR "entity extraction*" OR "file cluster*" OR "forward chaining" OR "generative AI" OR "generative adversarial network*" OR gan OR gans OR "hierarchical learning" OR "hyperparameter*" OR "knowledge discovery" OR "knowledge engineering" OR ( "learning" W/2 "algorithm" ) OR ( "learning" W/2 "scenario*" ) OR "linguistic analy*" OR "linguistic annotation*" OR "literature mining" OR "machine intelligence" OR ( "supervised" OR "unsupervised" ) W/2 "machine learning" OR "machine perception" OR "machine translation*" OR "natural language processing" OR nlp OR "large language model*" OR "computational tool*" OR "vector embedding" ) ) AND PUBYEAR > 2020 AND PUBYEAR < 2026

## Web of Science Core Collection

# Web of Science Search Strategy (v0.1)

# Database: Web of Science Core Collection

# Entitlements:

- WOS.IC: 1993 to 2025

- WOS.CCR: 1985 to 2025

- WOS.SCI: 1900 to 2025

- WOS.AHCI: 1975 to 2025

- WOS.BHCI: 2008 to 2025

- WOS.BSCI: 2008 to 2025

- WOS.ESCI: 2020 to 2025

- WOS.ISTP: 1990 to 2025

- WOS.SSCI: 1956 to 2025

- WOS.ISSHP: 1990 to 2025

# Searches:

1: ( TI= ( valid* OR reliabl* OR opportunit* OR challeng* OR risk* OR support* OR assst* OR perform* OR bias OR screening OR design* OR automi?ation OR reproducib* OR method* ) or AK=( valid* OR reliabl* OR opportunit* OR challeng* OR risk* OR support* OR assst* OR perform* OR bias OR screening OR design* OR automi?ation OR reproducib* OR method* ) ) Date Run: Fri Feb 14 2025 11:08:51 GMT+0000 (Greenwich Mean Time) Results: 8466400

2: TI=("meta-review*" OR "metareview*" OR "rapid review*" OR "scoping review*" OR "umbrella review*" OR "evidence review*" OR "literature review*" OR "systematic review*" OR "metaanalys*" OR "meta-analys*" OR ( ( evidence OR research ) near/4 synthe* ) ) or KP=("meta-review*" OR "metareview*" OR "rapid review*" OR "scoping review*" OR "umbrella review*" OR "evidence review*" OR "literature review*" OR "systematic review*" OR "metaanalys*" OR "meta-analys*" OR ( ( evidence OR research ) near/4 synthe* ) ) Date Run: Fri Feb 14 2025 11:09:38 GMT+0000 (Greenwich Mean Time) Results: 803584

3: TS= ( "active learning" OR ( "adaptive" near/2 "algorithm*" ) OR "artificial intelligence" OR ai OR "artificial narrow intelligence" OR ani OR ( "automat*" near/2 "tool*" ) OR "automat* learning" OR "automat* classification" OR "automat* term recognition" OR "backward chaining" OR "clustering engine*" OR "clustering tool*" OR "cognitive computing" OR "computational intelligence" OR "computational linguistic*" OR "computer reasoning" OR "computer vision" OR "connectionist model*" OR "data crunching" OR ( ( text OR data ) near/2 mining ) OR "datamining" OR "deep learning" OR "document cluster*" OR "entity annotation*" OR "entity extraction*" OR "file cluster*" OR "forward chaining" OR "generative AI" OR "generative adversarial network*" OR gan OR gans OR "hierarchical learning" OR "hyperparameter*" OR "knowledge discovery" OR "knowledge engineering" OR ( "learning" near/2 "algorithm" ) OR ( "learning" near/2 "scenario*" ) OR "linguistic analy*" OR "linguistic annotation*" OR "literature mining" OR "machine intelligence" OR ( "supervised" OR "unsupervised" ) near/2 "machine learning" OR "machine perception" OR "machine translation*" OR "natural language processing" OR nlp OR "large language model*" OR "computational tool*" OR "vector embedding" ) Date Run: Fri Feb 14 2025 11:09:50 GMT+0000 (Greenwich Mean Time) Results: 1190457

4: #1 AND #2 AND #3 Date Run: Fri Feb 14 2025 11:09:59 GMT+0000 (Greenwich Mean Time) Results: 2822

5: #1 AND #2 AND #3 and 2021 or 2022 or 2023 or 2024 or 2025 (Publication Years) Date Run: Fri Feb 14 2025 11:11:47 GMT+0000 (Greenwich Mean Time) Results: 2291

## Table S2: PRISMA-S Checklist

| **Section/topic** | **#** | **Checklist item** | **Location(s) Reported** |
| --- | --- | --- | --- |
| **INFORMATION SOURCES AND METHODS** | | | |
| Database name | 1 | Name each individual database searched, stating the platform for each. | Abstract, Methods Section, Supplementary File Part 2 |
| Multi-database searching | 2 | If databases were searched simultaneously on a single platform, state the name of the platform, listing all of the databases searched. | Supplementary File Part 2 |
| Study registries | 3 | List any study registries searched. | Not applicable |
| Online resources and browsing | 4 | Describe any online or print source purposefully searched or browsed (e.g., tables of contents, print conference proceedings, web sites), and how this was done. | Methods Section |
| Citation searching | 5 | Indicate whether cited references or citing references were examined, and describe any methods used for locating cited/citing references (e.g., browsing reference lists, using a citation index, setting up email alerts for references citing included studies). | Methods Section |
| Contacts | 6 | Indicate whether additional studies or data were sought by contacting authors, experts, manufacturers, or others. | Not applicable |
| Other methods | 7 | Describe any additional information sources or search methods used. | Methods Section |
| **SEARCH STRATEGIES** | | | |
| Full search strategies | 8 | Include the search strategies for each database and information source, copied and pasted exactly as run. | Supplementary File Part 2 |
| Limits and restrictions | 9 | Specify that no limits were used, or describe any limits or restrictions applied to a search (e.g., date or time period, language, study design) and provide justification for their use. | Methods section |
| Search filters | 10 | Indicate whether published search filters were used (as originally designed or modified), and if so, cite the filter(s) used. | Supplementary File Part 2 |
| Prior work | 11 | Indicate when search strategies from other literature reviews were adapted or reused for a substantive part or all of the search, citing the previous review(s). | Methods Section |
| Updates | 12 | Report the methods used to update the search(es) (e.g., rerunning searches, email alerts). | Not applicable |
| Dates of searches | 13 | For each search strategy, provide the date when the last search occurred. | Abstract, Methods Section, Supplementary File 2 |
| **PEER REVIEW** | | | |
| Peer review | 14 | Describe any search peer review process. | Not applicable |
| **MANAGING RECORDS** | | | |
| Total Records | 15 | Document the total number of records identified from each database and other information sources. | Results section (PRISMA flowchart), Supplementary File 2 |
| Deduplication | 16 | Describe the processes and any software used to deduplicate records from multiple database searches and other information sources. | Methods section |
|  |  |  |  |
| PRISMA-S: An Extension to the PRISMA Statement for Reporting Literature Searches Systematic Reviews | | |  |
| Rethlefsen ML, Kirtley S, Waffenschmidt S, Ayala AP, Moher D, Page MJ, Koffel JB, PRISMA-S Group. | | |  |
| Last updated February 27, 2020. | |  |  |

# Table S3: Alphabetized list of usable AI tools

| **Tool name** | **Hyperlink** |
| --- | --- |
| 3Ranker | <https://www.open3r.org/> |
| Abstrackr | <http://abstrackr.cebm.brown.edu/> |
| Anara | <https://anara.com/?from=unriddle> |
| Anne O'Tate | <https://arrowsmith.psych.uic.edu/cgi-bin/arrowsmith_uic/AnneOTate.cgi> |
| ASReview | <https://asreview.nl/> |
| BioTextQuest v2.0 | <http://bioinformatics.med.uoc.gr/shiny/biotextquest_kostas/> |
| Carrot tools: | <https://search.carrot2.org/> |
|  | Carrot Search: <https://carrotsearch.com/> |
|  | Lingo4G: https://carrotsearch.com/lingo4g/ |
|  | Lingo3G: <https://carrotsearch.com/lingo3g/> |
| Casper AI | [Casper AI chrome extension](https://chromewebstore.google.com/detail/casper-ai/fgfiokgecpkambjildjleljjcihnocel) |
| ChatGPT and ScholarGPT | <https://chatgpt.com/> |
|  | <https://chatgpt.com/g/g-kZ0eYXlJe-scholar-gpt> |
| ChatPDF | <https://www.chatpdf.com/> |
| Citationchaser | <https://estech.shinyapps.io/citationchaser/> |
| Claude | <https://claude.ai/> |
| Cochrane PICO annotator | <https://data.cochrane.org/pico-annotator/?#ajax/home.html> |
| Cochrane RCT classifier | [Cochrane RCT classifier - Screen4me workflow](https://training.cochrane.org/online-learning/publishing-cochrane-author-guidelines/screen4me) |
| Colandr | <https://www.colandrapp.com/> |
| Connected papers | <https://www.connectedpapers.com/> |
| Consensus.app | <https://consensus.app/> |
| Covidence | <https://www.covidence.org/> |
| Deduklick | <https://www.risklick.ch/deduklick> |
| DistillerSR and DistillerAI | <https://www.distillersr.com/> |
|  | <https://www.distillersr.com/products/distillersrai> |
| DocAnalyzer AI | <https://docanalyzer.ai/> |
| DoCTER | [icf DoCTER](https://www.icf-docter.com/#:~:text=DoCTER%2C%20ICF's%20Document%20Classification%20and,media%20messages%20%E2%80%94%20for%20expert%20review.) |
| Easy-peasy AI | <https://easy-peasy.ai/> |
| Elicit | <https://elicit.com/> |
| EPPI-reviewer | <https://eppi.ioe.ac.uk/cms/er4/> |
| ExaCT | [NRC Canada ExaCT demo](https://nrc.canada.ca/en/research-development/products-services/software-applications/exactdemo-clinical-information-extraction-system) |
| Gemini (previously known as Bard AI) | <https://gemini.google.com/> |
| Hipdf | <https://www.hipdf.com/> |
| Humata.ai | <https://www.humata.ai/> |
| iris.ai | <https://iris.ai/> |
| Jenni.ai | <https://jenni.ai/> |
| Laser AI | <https://www.laser.ai/> |
| Lateral | <https://www.lateral.io/> |
| Leximancer | <https://www.leximancer.com/> |
| LiteRev | <https://literev.unige.ch/> |
| Litmaps | <https://www.litmaps.com/> |
| LitStream | [https://www.icf.com/work/research-evaluation-surveys/litstream](https://www.icf.com/work/research-evaluation-surveys/litstream-systematic-literature-review) |
| LitSuggest | <https://www.ncbi.nlm.nih.gov/research/litsuggest/> |
| MeSHonDemand | [MeSHonDemand](https://meshb.nlm.nih.gov/MeSHonDemand?_gl=1*i8uyde*_ga*MTM5MTM2NjE3NC4xNzI3MDgyMDM2*_ga_7147EPK006*MTc0NTQxMDU0OS4yLjAuMTc0NTQxMDU0OS4wLjAuMA..*_ga_P1FPTH9PL4*MTc0NTQxMDU0OS4yLjAuMTc0NTQxMDU0OS4wLjAuMA..) |
| Microsoft Academic Graph | <https://www.microsoft.com/en-us/research/project/microsoft-academic-graph/> |
| Microsoft Copilot | <https://copilot.microsoft.com/> |
| Nested Knowledge and Robot Screener | <https://nested-knowledge.com/> |
|  | <https://about.nested-knowledge.com/docs/robot-screener/> |
| Openread | <https://www.openread.academy/> |
| pdf2gpt | <https://pdf2gpt.com/> |
| PICOportal | <https://picoportal.org/> |
| Pitts.ai | <https://pitts.ai/> |
| QDA Miner | <https://provalisresearch.com/products/qualitative-data-analysis-software/> |
| Rayyan | <https://www.rayyan.ai/> |
| RCT Tagger | <https://arrowsmith.psych.uic.edu/cgi-bin/arrowsmith_uic/RCT_Tagger.cgi> |
| Research Rabbit | <https://researchrabbitapp.com/> |
| Research Screener | <https://researchscreener.com/> |
| ResearchPal | <https://researchpal.co/> |
| revTools | <https://revtools.net/> |
| RobotAnalyst | <https://www.nactem.ac.uk/robotanalyst/> |
| SCISPACE | <https://scispace.com/> |
| Scite | <https://scite.ai/> |
| Semantic scholar | <https://www.semanticscholar.org/> |
| SRDB.PRO | <https://srdb.pro/default> |
| SWIFT-Active Screener | <https://www.sciome.com/swift-activescreener/> |
| SysRev | <https://www.sysrev.com/> |
| TERA tools (previously known as SR-Accelerator): | <https://tera-tools.com/> |
|  | <https://sr-accelerator.com/> |
| TextAnalyzer | <https://textanalyzer.org/lex/> |
| Trinka | <https://www.trinka.ai/> |
| Wordstat | <https://provalisresearch.com/products/content-analysis-software/> |

# Table S4: A complete list of open-source models and algorithms

| Model or algorithm | Hyperlink |
| --- | --- |
| MLScreener | <https://github.com/aaljaish/MLScreener> |
| FAST2 | <https://github.com/fastread/src> |
| SciBERT | <https://github.com/allenai/scibert> |
| BioBERT | <https://github.com/naver/biobert-pretrained> |
|  | <https://github.com/dmis-lab/biobert> |
| AMICA: Argument Mining In Covid-19 Articles | <https://github.com/francescoantici/amica?tab=readme-ov-file#amica-argument-mining-in-covid-19-articles> |
| slr-kit | <https://github.com/robolab-pavia/slr-kit> |
| KeyBERT | <https://maartengr.github.io/KeyBERT/> |
| SciFive | <https://github.com/justinphan3110/SciFive> |
| AlpaPICO | <https://github.com/shrimonmuke0202/AlpaPICO> |
| RoBERTabase and RoBERTalarge | <https://github.com/facebookresearch/fairseq> |
|  | <http://arxiv.org/abs/1907.11692> |
| PubMedBERT | <https://huggingface.co/microsoft/BiomedNLP-BiomedBERT-base-uncased-abstract-fulltext> |
| PAJO model | <https://github.com/xh621/PAJO-Deep-Learning-Model> |
| STEED | <https://osf.io/n8dz7/?view_only=> |
| NACSOS-nexus | <https://gitlab.pik-potsdam.de/mcc-apsis/nacsos/nacsos-core> |
|  | <https://arxiv.org/pdf/2405.04621> |
| BERTopic | <https://maartengr.github.io/BERTopic/index.html> |
| RMES | <https://data.mendeley.com/datasets/ccfnn3jb2x/1> |
| GAN-BioBERT | <https://zenodo.org/records/5699018> |
| BlueBERT | <https://github.com/ncbi-nlp/bluebert> |
| Longformer | <https://github.com/allenai/longformer> |
| TerMine | <https://www.nactem.ac.uk/software/termine/> |
| FusBERT | <https://github.com/rteb8/MSDS_FUSCapstone23/> |
| Zero shot classification | <https://github.com/carlosfmorenog/Zero-Shot_Abstract_Classification> |
| MedTrialExtractor | <https://github.com/TakedaGME/MedTrialExtractor/> |
| Clinical Trial Information Extractor | <https://doi.org/10.5281/zenodo.10419786> |
| Commercial General-Purpose Large Language Models for Evidence Synthesis | |
| Mistral AI | <https://mistral.ai/> |
| Meta’s Llama | <https://www.llama.com/> |
| Open AI’s GPT | <https://platform.openai.com/docs/models> |
| Anthropic’s Claude | <https://www.anthropic.com/> |
| Google’s Gemini | <https://deepmind.google/technologies/gemini/> |
| Moonshot AI | <https://www.moonshot.ai/> |
| M42’s Med42 | <https://huggingface.co/m42-health/med42-70b> |
| Google’s Flan T5 | <https://huggingface.co/google/flan-t5-xl> |
| Cohere’s CommandR+ | [https://docs.cohere.com/v2/docs/command-r-plus](https://docs.cohere.com/v2/docs/command-r-plus?utm_source=google&utm_medium=cpc&utm_campaign=fy26_emea_1_awareness_paidsearch_22295552317_179377625927_737093627746&utm_term=%7bquerystring%7d&gad_source=1&gbraid=0AAAAA-uE0d8XvylLOYuOOzmwQd4A0VDjP&gclid=Cj0KCQjw8cHABhC-ARIsAJnY12zAz-jcIaFTlof5wZGxb_0YKGk_aaJBRAYiPQhRbSiTt20d9KNr_X4aAne-EALw_wcB) |

# Table S5: Categorization of tools by evidence synthesis stage

| **Tool Name** | **Task in Evidence Synthesis** |
| --- | --- |
| ChatGPT | Multiple |
| Claude | Multiple |
| Colandr | Multiple |
| Covidence | Multiple |
| DistillerSR and DistillerAI | Multiple |
| Elicit | Multiple |
| EPPI-reviewer | Multiple |
| Gemini (Bard AI) | Multiple |
| Humata.ai | Multiple |
| iris.ai | Multiple |
| Litstream | Multiple |
| Microsoft Copilot | Multiple |
| Nested Knowledge and Robot Screener | Multiple |
| SCISPACE | Multiple |
| SRDB.PRO | Multiple |
| SysRev | Multiple |
| TERA | Multiple |
| 3Ranker | Search Tool |
| Anne O'Tate | Search Tool |
| BioTextQuest v2.0 | Search Tool |
| Carrot search | Search Tool |
| Citationchaser | Search Tool |
| Connected papers | Search Tool |
| Consensus.app | Search Tool |
| LiteRev | Search Tool |
| Litmaps | Search Tool |
| LitSuggest | Search Tool |
| MeSHonDemand | Search Tool |
| Microsoft Academic Graph | Search Tool |
| Openread | Search Tool |
| Research Rabbit | Search Tool |
| ResearchPal | Search Tool |
| ScholarGPT | Search Tool |
| Scite | Search Tool |
| Semantic scholar | Search Tool |
| Abstrackr | Screening Tool |
| ASReview | Screening Tool |
| Cochrane RCT classifier | Screening Tool |
| DoCTER | Screening Tool |
| Rayyan | Screening Tool |
| RCT Tagger | Screening Tool |
| Research Screener | Screening Tool |
| revTools | Screening Tool |
| RobotAnalyst | Screening Tool |
| SWIFT-Active Screener | Screening Tool |
| Casper AI | Data Extraction Tool |
| Cochrane PICO annotator | Data Extraction Tool |
| DocAnalyzer AI | Data Extraction Tool |
| Easy-peasy AI | Data Extraction Tool |
| ExaCT | Data Extraction Tool |
| Hipdf | Data Extraction Tool |
| pdf2gpt | Data Extraction Tool |
| PICOportal | Data Extraction Tool |
| Anara | Analysis Tool |
| Lateral.io | Analysis Tool |
| Leximancer | Analysis Tool |
| QDA Miner | Analysis Tool |
| TextAnalyzer | Analysis Tool |
| Wordstat | Analysis Tool |
| Jenni.ai | Writing Tool |
| Trinka | Writing Tool |
| ChatPDF | Data Extraction and Analysis Tool |
| Lingo3G and Lingo4G | Data Extraction and Analysis Tool |
| Pitts.ai | Data Extraction and Review Updates Tool |
| Deduklick | Deduplication Tool |
| Laser AI | Screening and Extraction Tool |
